# Transcriptome sequencing and miRNA-mRNA network construction in exosome of macrophage M2 in stomach adenocarcinoma

**DOI:** 10.1186/s12957-023-03070-1

**Published:** 2023-06-28

**Authors:** Dun Pan, Zhipeng Li, Xin Lin, Liangqing Li

**Affiliations:** 1 Department of Gastrointestinal Surgery, the First Affiliated Hospital, Fujian Medical University, Fuzhou, 350005 Fujian Province China; 2 Department of Gastrointestinal Surgery, National Regional Medical Center, Binhai Campus of the First Affiliated Hospital, Fujian Medical University, Fuzhou, 350212 China; 3 Fujian Research Institute of Abdominal Surgery, the First Affiliated Hospital, Fujian Medical University, Fuzhou, 350005 China

**Keywords:** Stomach adenocarcinoma, Exosome, Macrophage M2, RNA sequencing, miRNA, mRNA

## Abstract

**Background:**

Stomach adenocarcinoma (STAD) is the most common histological type of gastric cancer (GC). Macrophages are an essential part of the tumor microenvironment. We attempted to search for potential molecular markers associated with macrophages, which might be helpful for STAD diagnosis and treatment.

**Methods:**

Firstly, exosome in macrophages was extracted for RNA sequencing to identify differentially expressed microRNAs (miRNAs) (DEmiRNAs). Then, DEmiRNAs and differentially expressed mRNAs (DEmRNAs) were screened in the Cancer Genome Atlas (TCGA) database. The miRNAs related to macrophage M2 polarization were obtained by intersecting the DEmiRNAs obtained from the sequencing data and TCGA data. Using the Pearson correlation coefficient method, the mRNAs significantly related to macrophage M2 were screened out, followed by construction of the macrophage M2-miRNA-mRNA network. Subsequently, real-time-polymerase chain reaction (RT-PCR) and online datasets were applied to validate the expression of DEmiRNAs and DEmRNAs.

**Results:**

A total of 6 DEmiRNAs were identified in RNA sequencing; 59 DEmiRNAs and 1838 DEmRNAs were identified in TCGA database. Among which, a common miRNA (hsa-miR-133a-3p) associated with the M2 polarization of macrophages was identified. Fifteen common mRNAs were obtained between DEmRNAs and mRNAs targeted by DEmiRNAs. Eventually, a core macrophage M2-1 down-regulated miRNA-7 and up-regulated mRNAs network was constructed, including hsa-miR-133a-3p, SLC39A1, TTYH3, HAVCR2, TPM3, XPO1, POU2F1, and MMP14. The expression of miRNA and mRNAs was in line with the validation results of RT-PCR and online datasets.

**Conclusion:**

In this study, the screening of biomarkers in exosome of macrophage M2 may contribute to the prognosis of STAD patients.

**Supplementary Information:**

The online version contains supplementary material available at 10.1186/s12957-023-03070-1.

## Background

Stomach adenocarcinoma (STAD) is the most common histological type of GC [1]. Survival of STAD patients has improved dramatically over the past 20 years due to advances in treatment, but the prognosis remains suboptimal [2, 3]. Macrophages are an essential part of the tumor microenvironment (TME), and high infiltration of macrophages is related to poor prognosis in most tumors. Yue et al. [4] showed that the abundance of macrophages in STAD tissues was significantly higher than that in adjacent tissues, which was significantly associated with overall survival (OS). High abundance of macrophages is associated with poor prognosis in STAD patients. Macrophages are divided into two subgroups, M1 type macrophages (classically activated macrophages) and M2 type macrophages (alternately activated macrophages) according to their function and the secretion level of inflammatory factors [5]. Macrophages infiltrating into tumors play a “double-edged sword” role in the occurrence of tumors [6]. M1 macrophages can kill tumor cells, while M2 macrophages promote tumor growth [7]. The polarization of macrophages is closely related to the tumor microenvironment, mainly manifested as M2 macrophages, which is closely connected with tumor growth and development [8].

Abnormal expression of microRNAs (miRNAs) in tumor tissues can affect signaling pathways to produce tumor-promoting or suppressive effects [9]. MiRNAs are involved in the occurrence and progression of GC [10], which may have important implications for diagnosis and treatment. Exosomes are multivesicular bodies produced in cells, which can be produced by almost all types of cells [11]. Exosomes carry miRNAs, lncRNAs, circRNAs, mRNAs, and their degradation fragments involved in intracellular signal transduction and participate in the important regulation of cellular activities [12]. Exosomes play a crucial role in tumor metastasis, disease occurrence, and development [13]. As an important class of gene expression regulators, the in-depth study of the binding of miRNAs to exosomes has been widely concerned. Dou et al. showed that exosomal hsa-miR-27b-3p increased vascular permeability and promoted the development of colorectal cancer [14]. The study by Xu et al. showed that exosomal hsa-miR-139 could suppress GC progression and metastasis by reducing matrix metallopeptidase 11 (MMP11) in the tumor microenvironment [15].

In our study, we extracted exosomes from cultured macrophages and identified differentially expressed miRNAs (DEmiRNAs) associated with M2 polarization by RNA sequencing on the sequencing data M2 and M0 (naïve macrophage) using R package DESeq2. In addition, we screened DEmiRNAs and differentially expressed mRNAs (DEmRNAs) through the TCGA database. Finally, we selected macrophages M2-related DEmiRNAs and DEmRNAs to construct a macrophage.

M2-miRNA-mRNA network includes 8 candidate molecules (hsa-miR-133a-3p, SLC39A1, TTYH3, HAVCR2, TPM3, XPO1, POU2F1, and MMP14). These molecules may be served as the diagnostic and therapeutic targets for STAD.

## Methods

### Cell culture

The human monocyte leukemia cell line (THP-1) was first treated with 200 nM of propylene glycol methyl ether acetate (PMA) for 24 h to differentiate into macrophages. Cell adherence indicates successful induction. After culturing in PMA-free medium for 24 h, THP-1 macrophages were respectively treated with solvent and IL4 (20 ng/ml) + IL-13 (20 ng/ml) for 48 h in exosome-free medium. After 48 h of drug treatment, the cell supernatant was collected to extract exosomes.

### Markers expression of macrophages by real-time quantitative polymerase chain reaction (RT-qPCR)

The total RNA of THP-1 macrophages was extracted using Shanghai Pufei TRIzol kit, cDNA was obtained by reverse transcription using Promega M-MLV kit, and then RT-qPCR detection was used to detect gene expression. ACTB was used as an internal reference gene, and the relative expression levels of macrophage marker genes were determined by the 2-ΔΔCt method.

### Extraction of exosomes

The cell supernatant was thawed at 4 °C, centrifuged at 2000 g for 30 min at 4 °C, and filtered with a 0.22-µm filter. The filtrate was ultracentrifuged at 120,000 g 4 °C for 2 h, and the supernatant was carefully aspirated. The pellet was resuspended in the same volume of pre-cooled polybutylene succinate (PBS) and centrifuged at 120,000 g for 2 h at 4 °C. Aspirate the supernatant again, resuspend in 200 µL of cold PBS, and store at 4 °C. Exosomes were extracted by ultracentrifugation/kit method. M0 and M2 samples were subjected to transmission electron microscopy (TEM) and detection of protein marker (Western blot).

### Identification of exosomes

The exosome samples were first observed by TEM. A total of 20 µl of the exosome suspension was dropped on the fixed carbon mesh and left at room temperature for 20 min. The excess exosome suspension was carefully blotted dry with filter paper. A total of 20 µl of 2% phosphotungstic acid was dropped on the carbon mesh and left for 20 s. After blotting the excess phosphotungstic acid with filter paper, the carbon mesh was placed in a glass dish lined with filter paper and photographed by TEM.

For Western blot analysis, cell samples were washed twice with PBS, and an appropriate amount of radioimmune precipitation assay (RIPA) buffer was added to phenylmethylsulfonyl fluoride (PMSF). The samples were mixed by pipetting and lysed for 15 min on ice. The supernatant was taken to determine the protein concentration by bicinchoninic acid (BCA). New lysis buffer was added to adjust the protein concentration of each sample to 2 μg/μL. 6X of loading buffer was then added to samples and vortexed and stored at − 80 °C until analysis. A total of 20 µg of protein samples were loaded, separated on 10% of SDS-PAGE gels, and blotted on immunoblot polyvinylidene fluoride (PVDF) membranes and then blocked for 1 h and incubated overnight at 4 °C with primary antibody. Membranes were washed 4 times with Tris Buffer Solution Tween (TBST) and incubated with secondary antibody for 1.5 h at room temperature. Membranes were washed again and visualized using the enhanced chemiluminescence (ECL) method combined with X-ray films.

### Library construction, miRNA sequencing, and raw data processing

The total amount and fragment distribution of RNA in exosome samples were accurately detected by the highly sensitive Agilent 2100 pic600. After quality test, the Small RNA Sample Prep Kit was utilized to construct the library. Qubit2.0 was utilized for initial quantification. The insert size of the library was subsequently tested by Agilent 2100. The effective concentration of the library was accurately quantified by quantitative polymerase chain reaction (Q-PCR), and the different libraries were pooled according to the requirements of effective concentration and target data volume. The sequencing data were obtained through the SE50 strategy using an Illumina NovaSeq 6000 instrument. The original image data files obtained by sequencing were converted into sequenced reads using base calling analysis (raw reads). And raw reads were processed to obtain clean reads to ensure the quality of information analysis. Small RNA tags were mapped to reference sequence by Bowtie [16]. The above mapped reads on the reference sequence were aligned with the specified range sequence in miRBase to obtain detailed information about the sRNA matched to each sample. The correlation of miRNA expression among sequencing samples was analyzed by the cor function in R software.

### Identification of differentially expressed miRNAs (DEmiRNAs) in RNA sequencing

Differential expression analysis of miRNA was carried out between M2 and M0 by DESeq2 R package. The *p*-value was adjusted by the Benjamini and Hochberg method. *p* < 0.05 and |log fold change (FC)|> 1 were the screening criterion for DEmiRNAs.

### Identification of DEmiRNAs and differentially expressed mRNA (DEmRNAs) in the Cancer Genome Atlas (TCGA) database

RNA sequencing data (RPM value) of miRNA and the RNA sequencing data (FPKM value) of mRNA, from 372 cancer tissues and 32 normal tissues, were downloaded from the TCGA dataset. After data preprocessing, differential expression analysis was carried out with limma. DEmiRNAs and DEmRNAs were identified under the screening criteria of |logFC|> 1 and *p* < 0.05.

### Functional enrichment of DEmRNAs

The Gene Ontology (GO) and Kyoto Encyclopedia of Genes and Genomes (KEGG) functional enrichment analysis was performed by the DAVID database (https://david.ncifcrf.gov/tools.jsp) to explore potential function of DEmRNAs. The threshold value of *p* < 0.05 was the screening criterion.

### Distribution of immune cells

xCell is based on the single-sample gene set enrichment analysis (ssGSEA) method for calculating the distribution of immune cells in each sample. The xCell score of 64 immune cells in all samples was sorted into an immune cell infiltration matrix, and the immune cell types that differed between the two groups were calculated. The correlation between macrophage M2 and DEmRNAs was calculated by the Pearson correlation coefficient method.

### Construction of macrophage M2-miRNA-mRNA relationship network

The miRWalk (http://mirwalk.umm.uni-heidelberg.de/interactions/) was utilized to predict target mRNAs of miRNAs related to macrophage M2 polarization during gastric carcinogenesis. The relationship pairs have been validated in TargetScan, miRDB, and MiRTarBase. The predicted target mRNAs were intersected with the DEmRNAs. The correlation between macrophage M2 and core DEmRNAs and the miRNA-mRNA targeting relationship was combined to construct a macrophage M2-miRNA-mRNA network.

### Expression validation of DEmiRNAs and DEmRNAs by real-time-polymerase chain reaction (RT-PCR) and online datasets

Firstly, expression validation of DEmiRNAs and DEmRNAs was performed by RT-PCR in tumor tissues from STAD patients. Inclusion criteria for STAD patients were as follows: (1) The patients were initially diagnosed with STAD, (2) the patients did not undergo other therapy before diagnosis, (3) the patients had no other malignant tumor and other autoimmune diseases, and (4) the patients were 18 to 70 years old. The exclusion criteria for STAD patients were as follows: (1) patients had other malignancy, (2) patients received other treatment before surgery, (3) patients had incomplete clinical data, (4) patients had a history of STAD, and (5) patients with recurrence. Ten STAD patients were enrolled from First Affiliated Hospital of Fujian Medical University. Paracancerous samples and STAD exosome tissue samples were collected. Clinical data was registered in detail (Table 1), mainly including the age, sex, stage, grade, drinking, smoking, and family history. The Ethics Committee of First Affiliated Hospital of Fujian Medical University approved this study (2,020,219). Informed consent of patients and their families was obtained.

**Table 1:** Clinical information registry

Number	Age (years)	Sex	Height (cm)	Weight (kg)	BMI	Clinical stages			Stage	Histological subtype	Grade	Tumor metastasis	Alcohol history	Smoking history	Family history
						**T**	**N**	**M**							
1	54	Female	158	61	24.44	T4a	N2	M0	IIIB	Diffuse type	G3	No	No	No	No
2	72	Male	164	50	18.59	pT4a	N0	M0	IIB	Mixed type	G3	No	Yes	Yes	No
3	68	Male	165	56	20.57	T4a	N3	M0	IIIC	Diffuse type	G3	No	No	No	No
4	55	Male	176	77	24.86	T4a	N3	M0	IIIC	Diffuse type	G3	No	No	No	No
5	57	Female	160	54	21.09	T3	N0	M0	IIA	Diffuse type	G3	No	No	No	No
6	68	Male	170	70	24.22	T3	N0	M0	IIA	Intestinal type	G3	No	No	No	No
7	55	Female	170	73	25.26	T4a	N1	M0	IIIA	Diffuse type	G3	No	No	No	No
8	61	Female	155	42	17.48	T1a	N0	M0	IA	Diffuse type	G3	No	No	No	No
9	73	Male	169	63	22.06	T2	N0	M0	IB	Intestinal type	G3	No	No	No	No
10	70	Male	160	60	23.44	T2	N0	M0	IB	Diffuse type	G3	No	No	No	No

Total RNA was extracted from tissue samples by TRIzol® Reagent. FastKing cDNA first-strand synthesis kit (KR116) was applied to mRNA reverse transcription. miRNA first-strand cDNA synthesis (tailing) (B532451-0020) was applied to miRNA reverse transcription. RT-PCR was performed on mRNA with SuperReal PreMix Plus (SYBR Green) (FP205) and miRNA with miRNA qPCR Master Mix. Gene-9660 fluorescence quantitative PCR instrument was used for relative quantitative analysis of data by 2-^△△ct^ method. GAPDH, ACTB, and hsa-U6 were used as internal control for gene detection.

In addition, the GSE65801 dataset (tumor samples from 32 cases and 32 normal controls) and the GSE13911 dataset (tumor samples from 38 cases and 31 normal controls) were downloaded from Gene Expression Omnibus (GEO) to validate the expression of core DEmRNAs by a rank-sum test.

### Drug prediction of core DEmRNAs

Through the DGIdb database (Version: 3.0.3, http://www.dgidb.org/), core DEmRNAs related to macrophage M2 polarization were searched for potential drugs targets for GC. The identified target network was visualized by Cytoscape software [17].

### Statistical analysis

The correlation of miRNA expression between sequencing samples was analyzed by the cor function in R software. The differential expression analysis was performed by DESeq2 and limma. The functional enrichment analysis was performed using the DAVID database. Targeting relationship prediction was performed using miRWalk. *p* < 0.05 was considered as statistical significance.

## Results

### Markers expression of macrophages

We assessed the successful differentiation of macrophages by measuring the expression of two M1 and M2 markers, respectively. Among them, TNF and NOS2 are markers of M1 macrophages, PPARG and CCL22 are markers of M2 macrophages, and both M1 and M2 markers were significantly expressed (Supplementary Fig. 1).

### Identification of exosomes

**Fig. 1:**
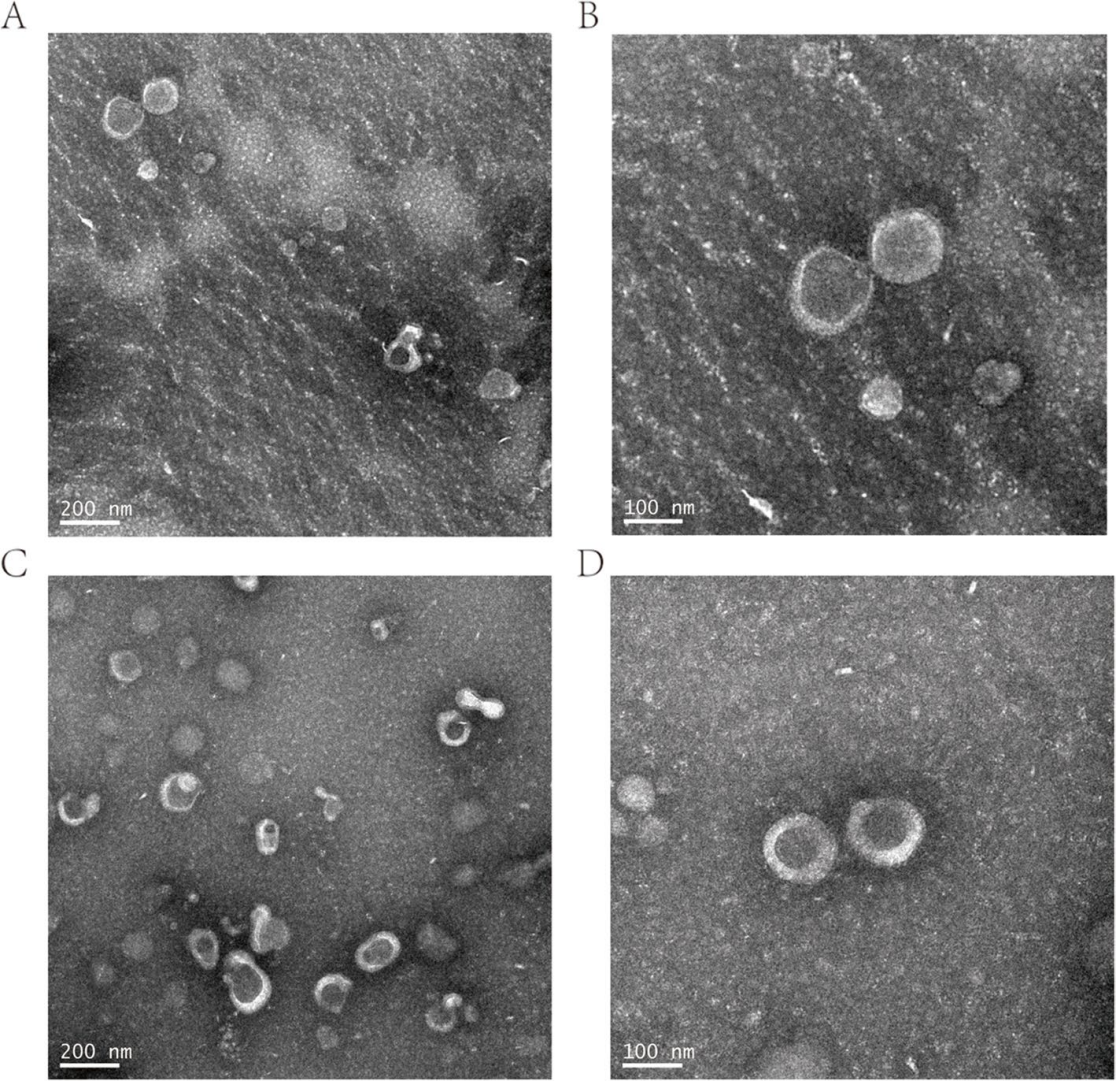
Transmission electron micrographs of exosomes. **A**, **B** The view of TEM displayed the cup-shaped morphology of M0 exosomes. Scale bar, 200 nm (**A**). Scale bar, 100 nm (**B**). **C**,** D** The view of TEM displayed the cup-shaped morphology of M2 exosomes. Scale bar, 200 nm (**C**). Scale bar, 100 nm (**D**)

**Fig. 2:**
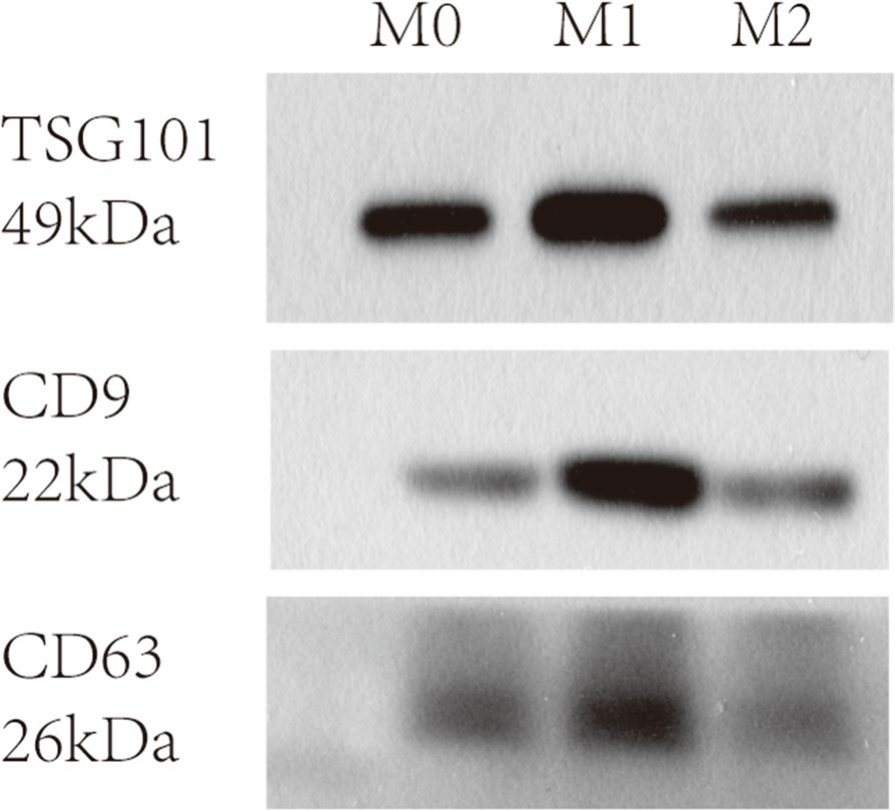
Western blot analysis for exosome markers TSG101, CD9, and CD63

The samples were photographed under the electron microscope with a clear field of view and complete shape. The view of TEM displayed the cup-shaped morphology of exosomes (Fig. 1). Our initial assumption was to search for miRNAs at the common intersection of M0, M1, and M2. However, there were no M1-related miRNAs in the intersection set. Therefore, the data of two experimental samples M0 and M2 were finally selected. Western blot analysis detected the expression of biomarkers of exosomes, including TSG101, CD9, and CD63 (Fig. 2), which further confirmed the TEM image data. Therefore, the extraction, identification, and detection of exosomes from M0 and M2 samples met the standards for exosome detection.

### Screening of DEmiRNAs and DEmRNAs in STAD

**Fig. 3:**
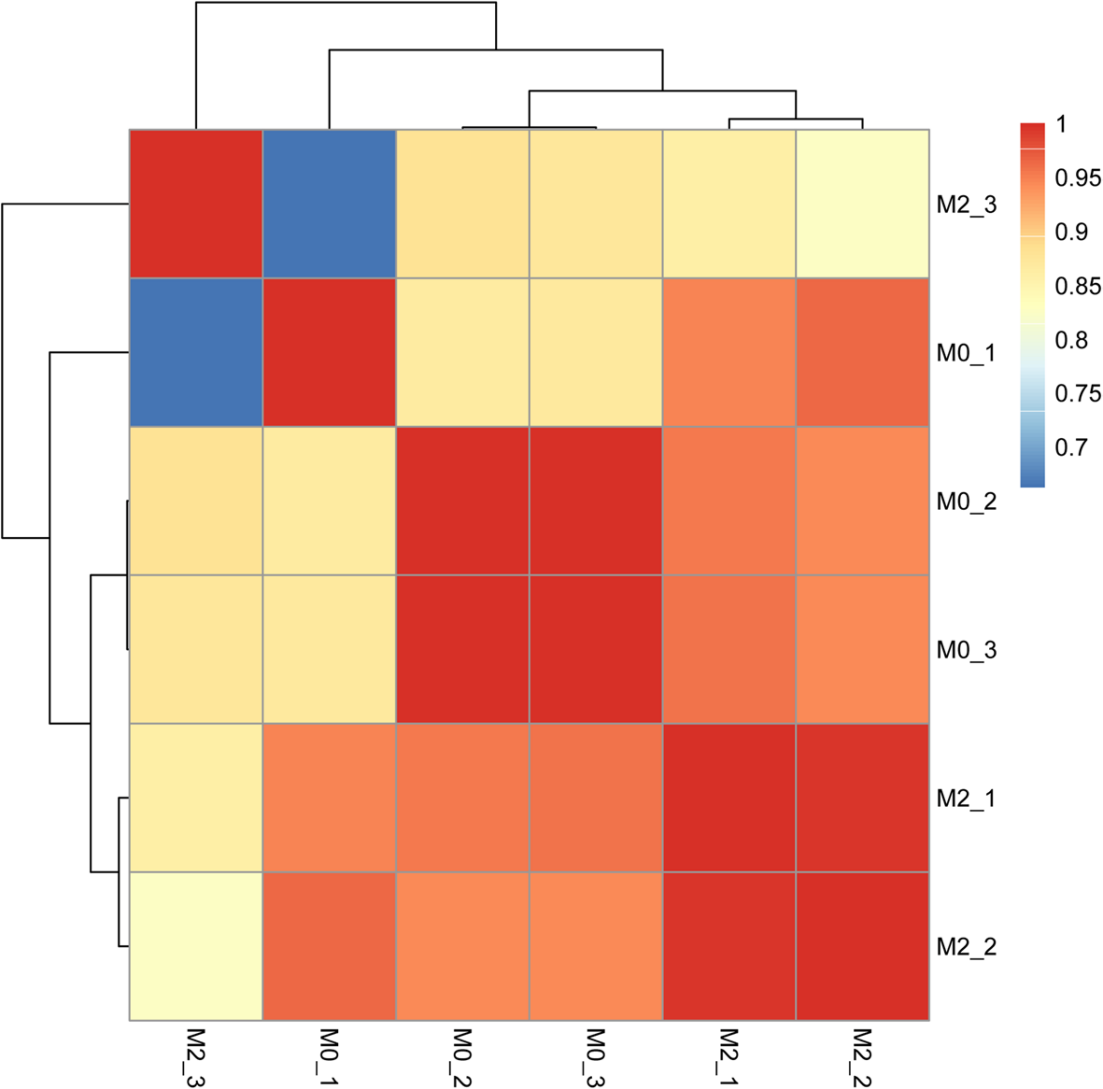
Heat map of miRNA expression correlation among samples. The darker the red color, the higher the similarity between samples, and the darker the blue, the lower the the similarity between samples

**Fig. 4:**
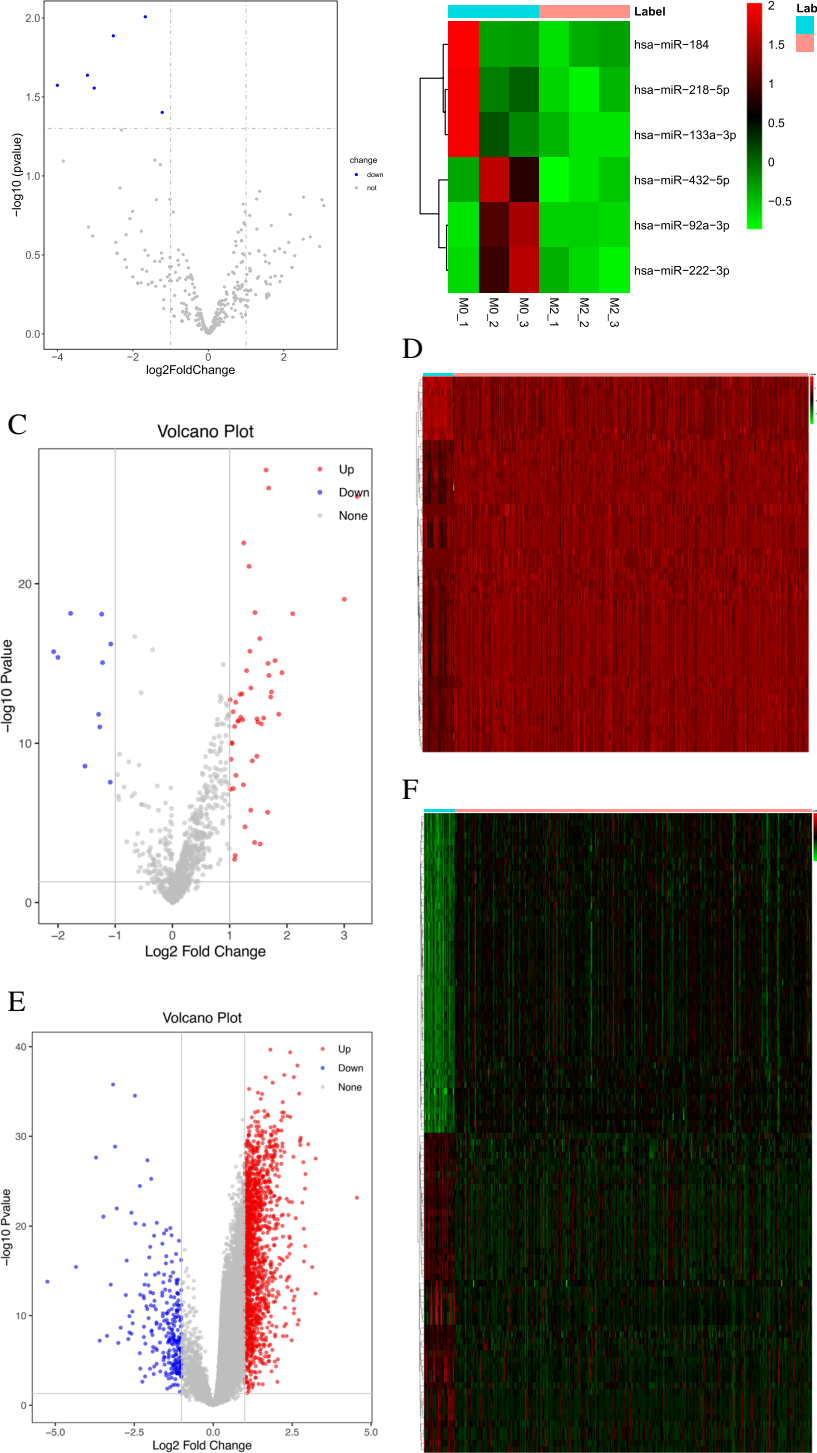
Volcano plots and heat maps of DEmiRNAs and DEmRNAs in STAD. **A**, **B** DEmiRNAs in the RNA sequencing. **C**, **D** DEmiRNAs in the TCGA dataset. **E**, **F** DEmRNAs in the TCGA dataset. Note: In the volcano plot, blue indicated down-regulated gene, red indicated up-regulated gene, and gray indicated not-significant gene. In the heat maps, each small square represents each gene, and its color represents the expression level of the gene. The greater the expression level, the darker the color (red means upregulation, green means downregulation). Each row represents the expression level of each gene in different samples, and each column represents the expression level of all genes in each sample

The closer the correlation coefficient is to 1, the higher the similarity of expression patterns between samples. We can observe that our samples have extremely high correlations (Fig. 3). A total of 6 DEmiRNAs were obtained in RNA sequencing (Fig. 4A, B). Interestingly, all these miRNAs were downregulated. In total, 59 DEmiRNAs were examined in the TCGA dataset, including 49 up-regulated and 10 down-regulated (Fig. 4C, D). A down-regulated miRNA (hsa-miR-133a-3p) associated with M2 polarization of macrophages during gastric carcinogenesis was obtained by intersecting DEmiRNAs in RNA sequencing and TCGA dataset. In addition, 1838 DEmRNAs were obtained, including 1586 up-regulated and 252 down-regulated (Fig. 4E, F). In total, 15 mRNAs were identified by intersecting the DEmRNAs and predicted negatively regulated target mRNAs of hsa-miR-133a-3p, including DNA cross-link repair 1A (DCLRE1A), solute carrier family 39 member 1 (SLC39A1), erb-b2 receptor tyrosine kinase 2 (ERBB2), BCL2 like 1 (BCL2L1), tweety family member 3 (TTYH3), hepatitis A virus cellular receptor 2 (HAVCR2), tropomyosin 3 (TPM3), replication factor C subunit 3 (RFC3), exportin 1 (XPO1), POU class 2 homeobox 1 (POU2F1), myelin regulatory factor (MYRF), matrix metallopeptidase 14 (MMP14), polypyrimidine tract binding protein 3 (PTBP3), SPT16 homolog, facilitates chromatin remodeling subunit (SUPT16H), and TBL1X/Y related 1 (TBL1XR1).

### Functional analysis of DEmRNAs in STAD

**Fig. 5:**
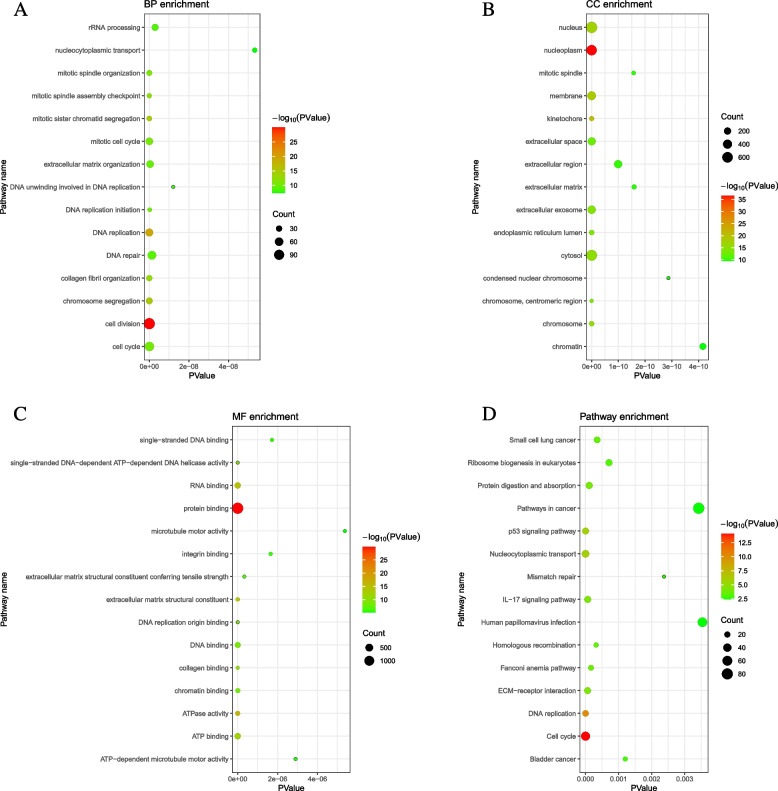
GO and KEGG enrichment results of DEmRNAs in STAD. **A** Biological process (BP). **B** Cellular component (CC). **C** Molecular function (MF)

Top 15 enrichment functions for biological process (BP), cellular component (CC), molecular function (MF), and KEGG are shown in Fig. 5A–D, respectively. In the aspect of BP, DEmRNAs were involved in cell division, DNA replication, and cell cycle (Fig. 5A). In terms of CC, DEmRNAs were involved in nucleoplasm, nucleus, and cytosol (Fig. 5B). In the MF, DEmRNAs were involved in protein binding, RNA binding, and ATP binding (Fig. 5C). KEGG analysis results showed the DEmRNAs mainly involve in cell cycle, DNA replication, and pathways in cancer (Fig. 5D). Additionally, XPO1 and POU2F1 were involved in human T-cell leukemia virus 1 and lipid and atherosclerosis signaling pathways, respectively.

### Macrophage M2 correlation analysis and construction of macrophage M2-miRNA-mRNA relationship network in STAD

**Fig. 6:**
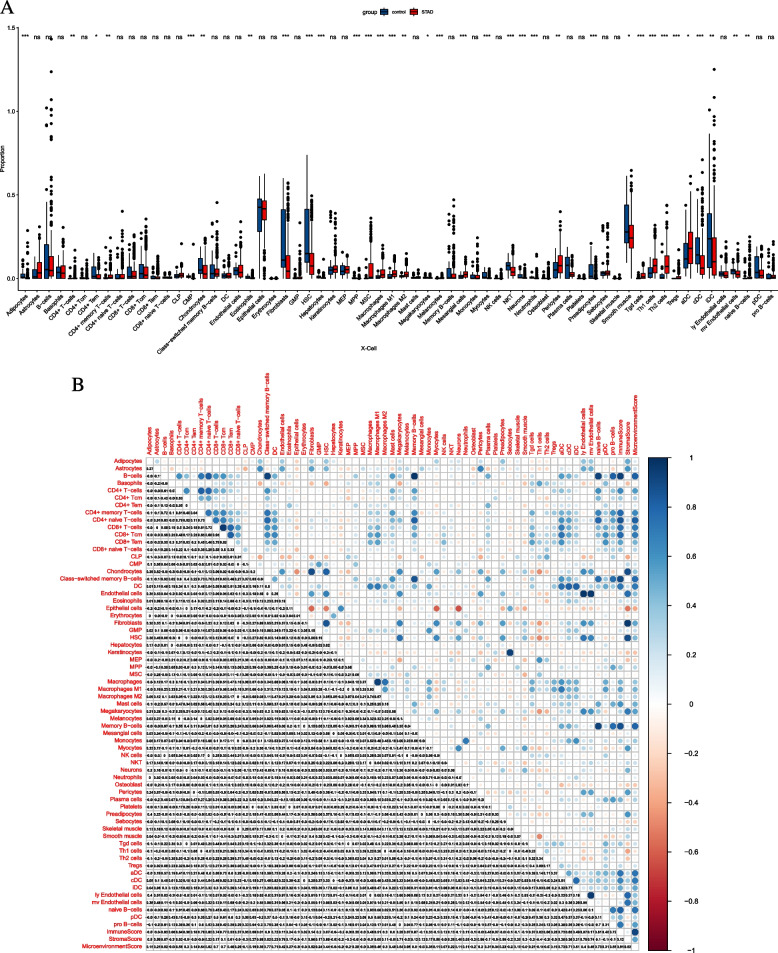
Differences in the proportion of 64 immune cells infiltrating between normal tissue and tumor tissue (**A**) and the correlation matrix of tumor-infiltrating immune cells (**B**) in STAD. *p* < 0.05*, *p* < 0.01**, *p* < 0.001***, ns: not significant

**Fig. 7:**
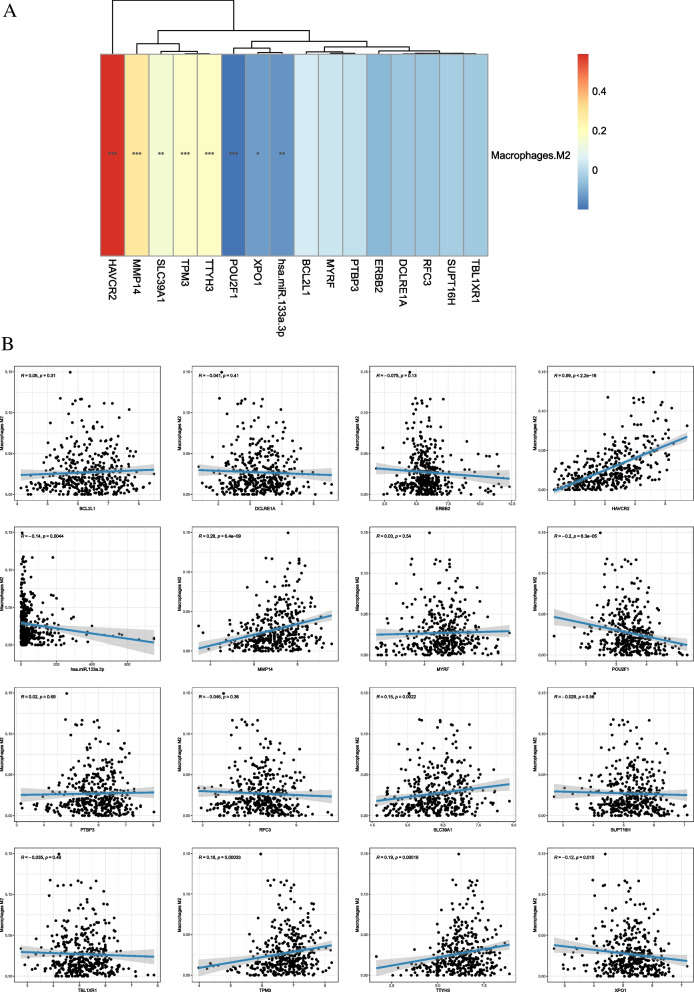
Heat maps (**A**) and scatter plots (**B**) of correlation between DEmRNAs and macrophage M2 in STAD. *p* < 0.05*, *p* < 0.01**, *p* < 0.001***

**Fig. 8:**
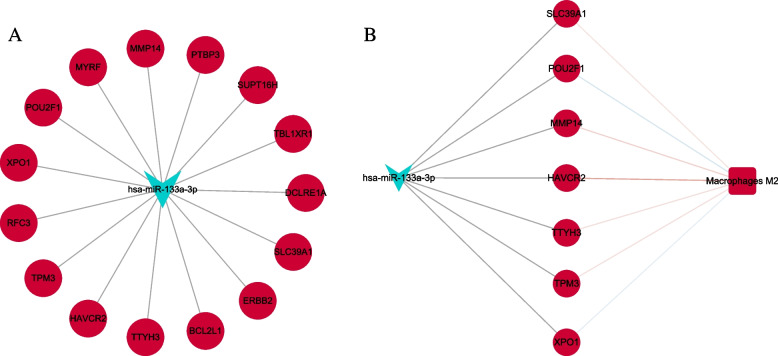
The miRNA-mRNA network (**A**) and macrophage M2-miRNA-mRNA network (**B**). All points are DEmiRNAs (V shaped), DEmRNAs (circles), macrophage M2 (rectangles). Red and green represent upregulation and downregulation, respectively

Infiltrated immune cells are used as biomarkers of response to immunotherapy in many cancers [18]. The distribution of 64 immune cells was calculated and found that the proportion of macrophages infiltrated in tumor tissue was much higher than that of normal tissue (Fig. 6A). It is indicated that macrophages are involved in the occurrence and progression of cancer. It is noted that the infiltration degree of macrophage M2 is high in STAD. Immune cell correlation analysis revealed that macrophage M2 was positively correlated with the majority of immune cells (Fig. 6B). The correlation between macrophage M2 and 15 DEmRNAs was calculated using Pearson’s correlation coefficient method. A total of 7 up-regulated mRNAs were significantly correlated with macrophage M2, including SLC39A1, TTYH3, HAVCR2, TPM3, XPO1, POU2F1, and MMP14 (Fig. 7A–B). The hsa-miR-133a-3p-15 DEmRNAs network was presented in Fig. 8A. Based on macrophage M2, 1 miRNA (hsa-miR-133a-3p), and 7 mRNAs, the core macrophage M2-miRNA-mRNA network was constructed (Fig. 8B).

### Expression validation of DEmiRNAs and DEmRNAs

**Fig. 9:**
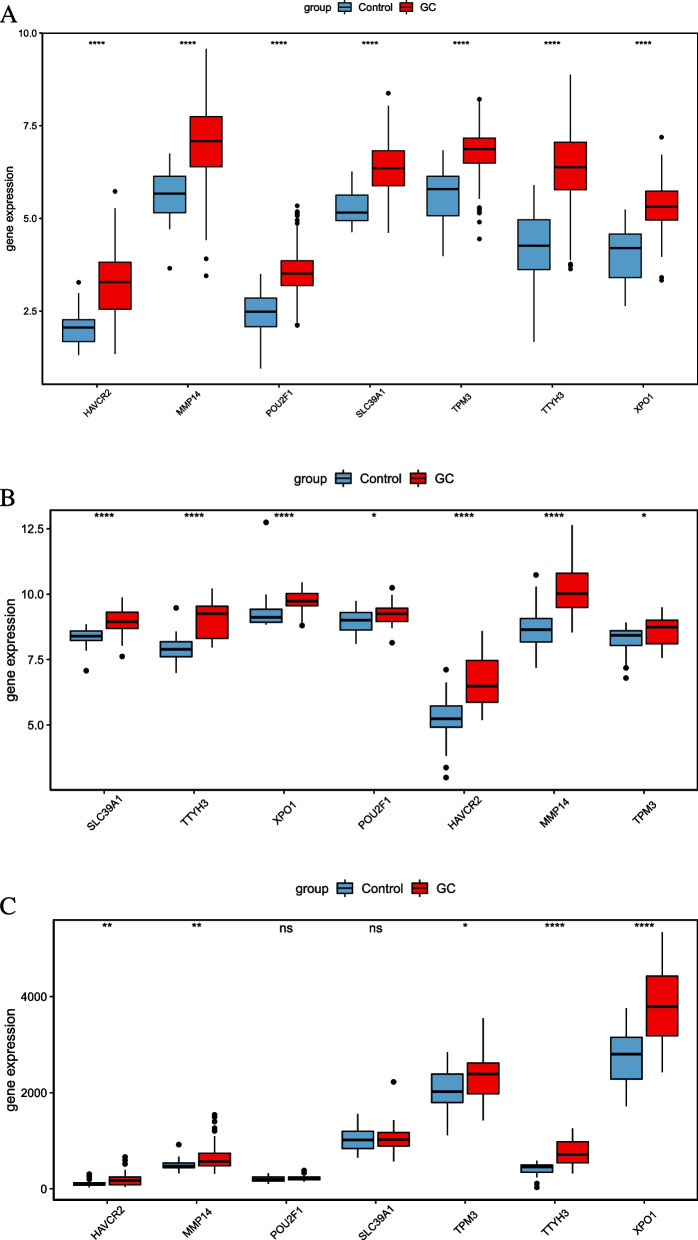
Expression validation of macrophage M2 polarization-related DEmiRNAs and DEmRNAs. **A** In TCGA dataset. **B** In GSE65801 dataset. **C** In GSE13911 dataset. *p* < 0.05*, *p* < 0.01**, *p* < 0.001***, *p* < 0.0001****, ns not significant

In this study, SLC39A1, TTYH3, HAVCR2, TPM3, XPO1, POU2F1, MMP14, and hsa-miR-133a-3p, macrophage M2-related molecules, were selected for RT-PCR verification (supplementary Fig. 2). TTYH3 was significantly over expressed in STAD patients (supplementary Fig. 2A). HAVCR2, SLC39A1, XPO1, and POU2F1 tend to be up-regulated in STAD patients (supplementary Fig. 2B–E). TPM3 and MMP14 showed no obvious expression trend (Supplementary Fig. 2F–G), and hsa-miR-133a-3p was downregulated in STAD patients (Supplementary Fig. 2H). In addition, expression of 7 mRNAs associated with macrophage M2 polarization was validated in GSE65801and GSE13911 datasets (Fig. 9A, B). We can find that mRNA expression was upregulated in the GC group. This is consistent with the expression in the TCGA dataset (Fig. 9C).

### Drug prediction of core DEmRNAs

**Fig. 10:**
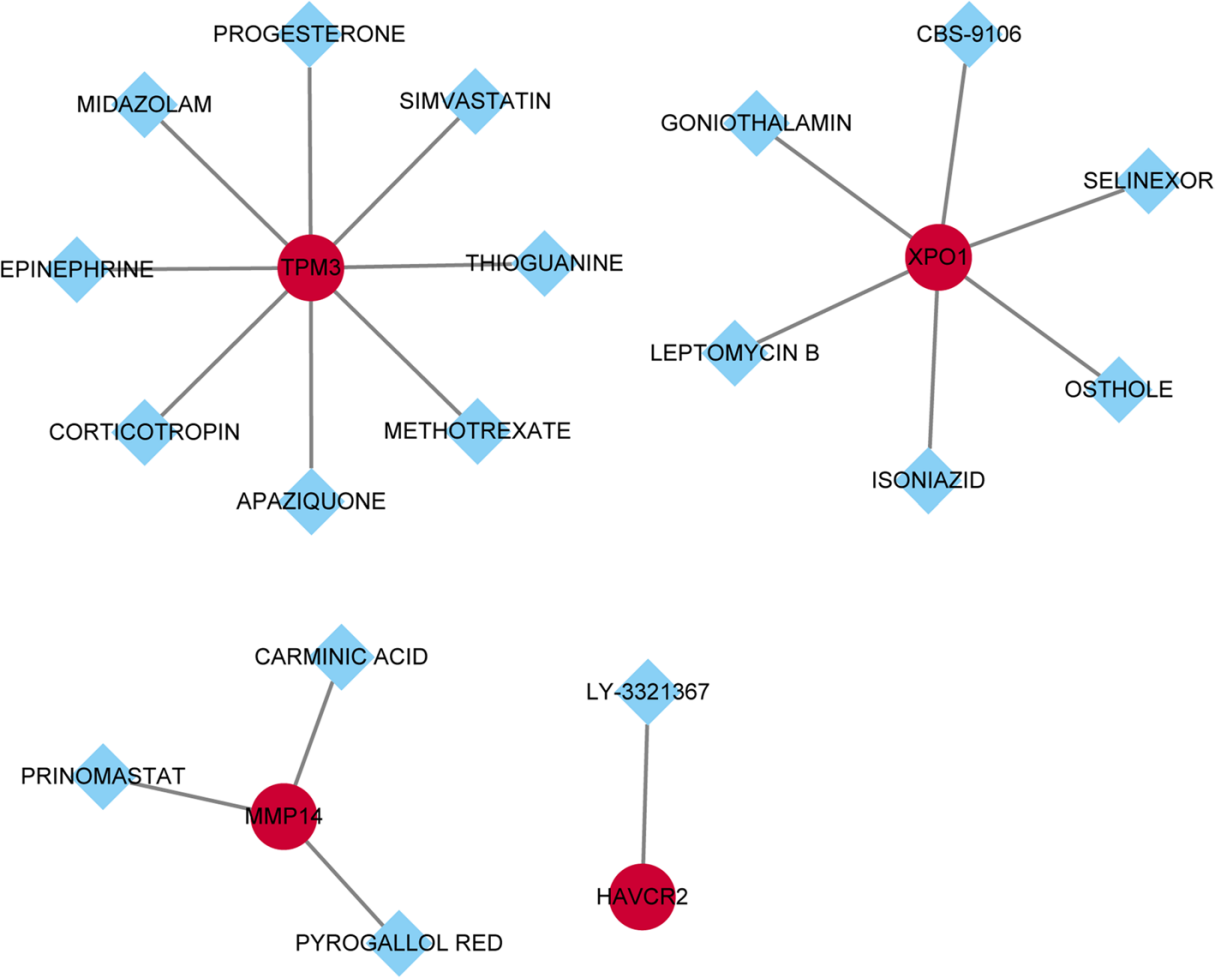
Drug-genes interaction. Red nodes represent up-regulated macrophage M2 polarization-related genes; blue diamonds represent drugs

In the DGIdb database, 4 DEmRNAs (TPM3, XPO1, MMP14, and HAVCR2) were screened out to be targets of 18 drugs for the treatment of GC (Fig. 10).

## Discussion

GC is a common malignancy [19], causing great harm to global health. STAD accounts for 95% of gastric malignancy [20] and is prone to invasion and metastasis [21]. Since the majority of patients with STAD are diagnosed at a late stage [22], early diagnosis and treatment are crucial for prolonging the survival in patients with STAD [23].

Macrophages are an important cell type in the immune system involved in the progression of various cancers [24]. Macrophages are classified into tumor-suppressive M1 type (activated by interferon-γ (IFN-γ) stimulation) and tumor-promoting M2 type (activated by IL-4 and IL-13 stimulation) according to the type of stimulation from their environment [25]. Macrophages can secrete exosomes carrying drug-resistant molecules and transfer drug-resistant molecules through the interaction of exosomes in the tumor microenvironment, thereby enhancing tumor cell resistance to drugs [26]. The study by Valadi, Ekström, Bossios, Sjöstrand, Lee, and Lötvall found that cellular exosomes are mediators of intercellular communication within the tumor microenvironment [12]. Zheng et al. demonstrated that exosomes derived from M2 macrophages can promote GC cell migration [27]. Therefore, the study of targeting exosomes has become a new hotspot in cancer research.miRNAs represent one of the major RNAs contained in exosomes for regulating the expression of complementary mRNAs [28]. In the new era of cancer treatment, miRNAs are expected to be used for early diagnosis and treatment of patients to prolong their survival [29, 30]. In our study, we screened 1 miRNA (hsa-miR-133a-3p) related to M2 and predicted 7 negatively targeted mRNAs (SLC39A1, TTYH3, HAVCR2, TPM3, XPO1, POU2F1, and MMP14) to construct a macrophage M2-miRNA-mRNA network.

Hsa-miR-133a-3p is one of the most frequently down-regulated miRNAs in numerous human malignancies, suggesting it may play a key role in tumor progression in numerous malignancies, such as colorectal cancer [31] and esophageal cancer [32]. Wang et al. [33] found that hsa-miR-133a-3p was downregulated in both adenoma and cancer tissues, which provided a potential early miRNA marker for colorectal cancer screening. Li et al. [34] screened a variety of GC-related miRNAs from the TCGA database, randomly selected to demonstrate the expression of some key miRNA expression and found that hsa-miR-133a-3p was downregulated in GC patients. In this study, we detected hsa-miR-133a-3p was downregulated in STAD tissues by detection of exosomes in M2 macrophages for the first time. We speculate the differential expression of hsa-miR-133a-3p may be involved in immune-related mechanisms, especially the difference with the M2 phenotype of macrophages, which may serve as a theoretical basis for the subsequent screening of immunotherapy targets.

SLC39A1, a zinc ion transporter located in the plasma membrane, has zinc uptake activity [35, 36] and exhibits oncogenic properties in a variety of malignancies. The study by Wang et al. [37] demonstrated that SLC39A1 was upregulated in glioma tissues, and high SLC39A1 expression predicted poor survival. Ma et al. [38] demonstrated that SLC39A1 was overexpression in hepatocellular carcinoma, speculating that SLC39A1 is an unfavorable prognostic biomarker for hepatocellular carcinoma. Ding et al. [39] analyzed the prognostic value of SLC family members in GC and explored their correlation with survival outcomes of GC patients. SLC39A1 in GC tissues was significantly higher than that in normal tissues, and GC patients had poorer OS. In our study, the validation results showed that SLC39A1 was also highly expressed in cancer tissues, which indicate the role of SLC39A1 in the development of STAD.

Several previous studies have shown that TTYH3 is over expressed in cancer, indicating poor cancer prognosis. The team of Polash Kumar Biswas showed that the expression of TTYH3 was higher in bladder cancer [40] and GC [41] than in normal tissues, which was significantly associated with reduced patient survival, suggesting that TTYH3 may be a therapeutic target. In this study, we found that TTYH3 was upregulated in STAD. Overall, TTYH3 may be a potential prognostic marker for GC patients.

HAVCR2 encodes the TIM-3 protein, a potential immune checkpoint target in tumors [42]. Several studies have shown that HAVCR2 is significantly upregulated in clear cell renal carcinoma [43], bladder urothelial carcinoma [44], and STAD [45] and is associated with poor prognosis in cancers. In addition, Li et al. [46] found that HAVCR2 expression was significantly correlated with pan-cancer prognosis, immune cell infiltration, and immune-related markers. In this study, HAVCR2 was also a targeted gene of hsa-miR-133a-3p, further suggesting that HAVCR2 may be a key regulatory role in the progression of STAD.

Numerous studies have shown that TPM3 promotes tumor cell metastasis. Amplification and overexpression of TPM3 are observed in hepatocellular carcinomas. Cui et al. [47] demonstrated that knockdown of TPM3 markedly inhibited the migration and invasion of hepatocellular carcinomas cells. The study by Chen et al. [48] showed that TPM3 can promote the proliferation, migration, and metastatic potential of esophageal squamous cell carcinoma cells (ESCC), which may become a new indicator of prognosis in patients with ESCC. Our study further confirmed that high expression of TPM3 means poor prognosis in STAD.

XPO1 is a major nuclear exporter of many tumor suppressor and chemotherapeutic targets [49, 50], and overexpression of XPO1 has been shown to be relative to poor prognosis or resistance to chemotherapy in various cancers [51]. Rachel Sexton et al. [52] found that XPO1 was over expressed in cancer cells compared to normal tissues, demonstrating that XPO1 is an effective therapeutic target for GC. Herein, our study found that XPO1 was involved in the human T-cell leukemia virus 1 signaling pathway. It has been proposed that HTLV-1 may be involved in the development of gastric T-cell lymphoma [53], which may reveal the key and possible mechanism of action of XPO1 in STAD.

POU2F1, a multifunctional transcription factor, promotes tumorigenesis and progression by regulating tumor-specific gene expression. For example, POU2F1 promotes the growth and metastasis of hepatocellular carcinoma through the FAT1 signaling pathway [54]. POU2F1 promotes GC cell viability and tumor growth via transcriptional activation of lncRNA TTC3-AS1 [55], demonstrating that POU2F1 is highly expressed in GC patients and predicts poor prognosis. In the present study, KEGG analysis showed POU2F1 was involved in lipid and atherosclerosis signaling pathway. Low-density lipoprotein accumulation is highly correlated with the development of cancer and cardiovascular disease [56]; there may be a link between cardiovascular disease and cancer.

MMP14 is a metalloproteinase involved in angiogenesis and cancer invasion. MMP14 is over expressed in cancer tissues and may be involved in tumor progression of GC. Dong et al. [57] showed that MMP14 was over expressed in GC tissue and was significantly correlated with clinical stage and distant metastasis. Wang et al. [58] revealed that high MMP14 expression was related to worse GC prognosis based on database validation analysis. In conclusion, MMP14 plays a major role in the progression and prognosis of GC and can be served as a biomarker to judge the prognosis of GC patients.

In addition, related drugs were searched for genes related to macrophage M2 polarization. Among them, simvastatin exerts antitumor effects on GC through impairing cell proliferation and caspase-3/GSDME-mediated pyroptosis [59]. Oral selinexor significantly inhibits tumor growth in GC xenograft models [60]. Osthole inhibits GC cell cycle arrest by downregulating PI3K/Akt signaling pathway, thereby exerting potential anti-GC effects [61]. It shows that the drugs have a potential therapeutic effect on GC.

However, there are limitations to our study. The sample size of RT-PCR experiments is small, and more samples are needed to verify the data. The specific molecular mechanism of the identified molecules in STAD is still unclear, and extensive experiments are needed to further study. Despite the limitations of the study, our findings may provide new clews for further research in STAD.

## Conclusions

Collectively, we screened 1 miRNA (in exosome) and 7 mRNAs related to M2, revealing that they may be potential diagnostic biomarkers in STAD. This study can serve as a theoretical basis for the diagnosis and development of STAD patients.

## Supplementary Information

**Additional file 1: Supplementary Fig. 1.** Expression of representative M1 and M2 marker genes of M1 and M2 THP-1 macrophage. A: The expression of M1 marker gene TNF; B: The expression of M1 marker gene NOS2; C: The expression of M2 marker gene PPARG; D: The expression of M2 marker gene CCL22. Gene expression (2-△△ct) is relative to M0 macrophages.

**Additional file 2: Supplementary Fig. 2.** Expression validation of macrophage M2 polarization-related DEmiRNA and DEmRNAs in RT-PCR.

**Additional file 3.** TSG101, CD9, and CD63.

## Data Availability

All data generated or analyzed during this study are included in this published article. STAD-miRNA (https://gdc-hub.s3.us-east-1.amazonaws.com/download/TCGA-STAD.mirna.tsv.gz) and STAD-mRNA (https://gdc-hub.s3.us-east-1.amazonaws.com/download/TCGA-STAD.htseq_fpkm.tsv.gz) were downloaded from TCGA (https://tcga-data.nci.nih.gov/tcga/) database. GSE65801 dataset (https://www.ncbi.nlm.nih.gov/geo/query/acc.cgi?acc=GSE65801) and GSE13911 dataset (https://www.ncbi.nlm.nih.gov/geo/query/acc.cgi?acc=GSE13911) were downloaded from the GEO database (https://www.ncbi.nlm.nih.gov/geo/). The raw data of sequencing are available from the corresponding author on reasonable request.
