# Supplementary figures and images for: Transcriptome sequencing and miRNA-mRNA network construction in exosome of macrophage M2 in stomach adenocarcinoma
Source: World J Surg Oncol. 2023 Jun 28;21:193. doi: 10.1186/s12957-023-03070-1 (PMC10304264; doi:10.1186/s12957-023-03070-1)

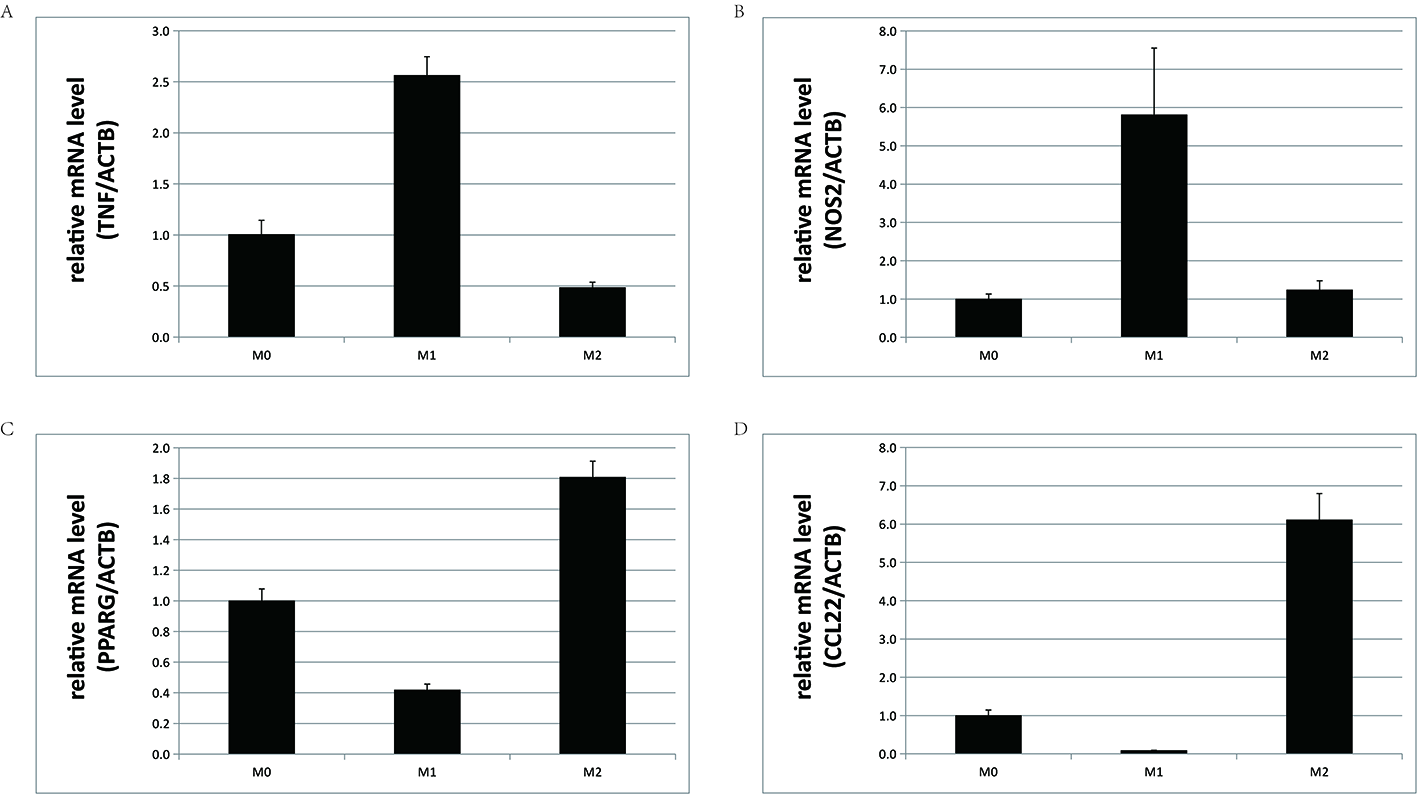

Supplement: Supplementary file 1 — Additional file 1: Supplementary Fig. 1. Expression of representative M1 and M2 marker genes of M1 and M2 THP-1 macrophage. A: The expression of M1 marker gene TNF; B: The expression of M1 marker gene NOS2; C: The expression of M2 marker gene PPARG; D: The expression of M2 marker gene CCL22. Gene expression (2-△△ct) is relative to M0 macrophages. [file 12957_2023_3070_MOESM1_ESM.tif]

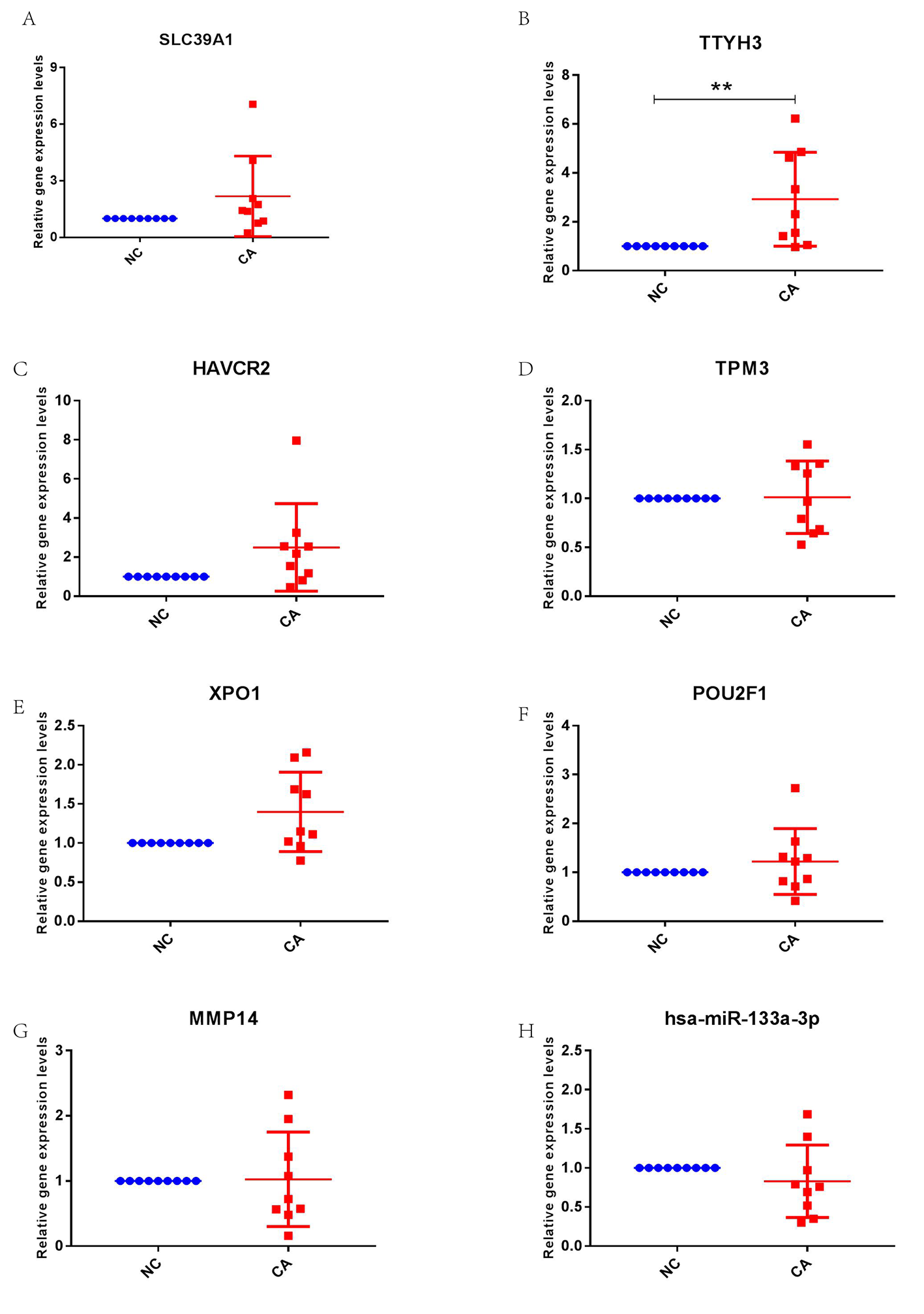

Supplement: Supplementary file 2 — Additional file 2: Supplementary Fig. 2. Expression validation of macrophage M2 polarization-related DEmiRNA and DEmRNAs in RT-PCR. [file 12957_2023_3070_MOESM2_ESM.tif]

TSG101


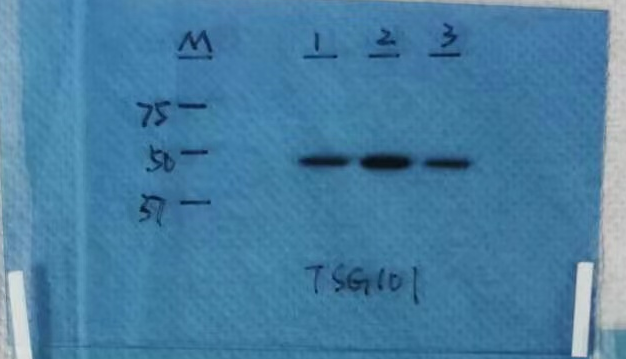


CD9


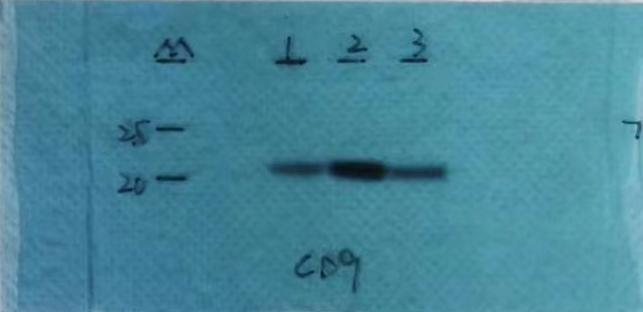


CD63


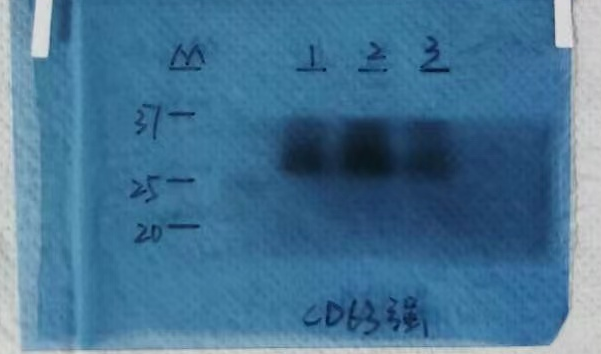

Supplement: Supplementary file 3 — Additional file 3. TSG101, CD9, and CD63. [file 12957_2023_3070_MOESM3_ESM.docx]
